# Thrombosis – Besieged but Poorly Understood

**DOI:** 10.3389/fcvm.2014.00004

**Published:** 2014-08-18

**Authors:** Irene M. Lang

**Affiliations:** ^1^Division of Cardiology, Department of Internal Medicine II, Vienna General Hospital, Medical University of Vienna, Vienna, Austria

**Keywords:** thrombosis, vascular biology, myocardial infarction, stroke, venous thromboembolism

In biology, thrombosis is a mechanism of maintaining the integrity of biological surfaces in higher mammals, limiting fluid loss, and facilitating the recognition, containment, and destruction of pathogens and foreign materials by a scaffold of fibrin and DNA strands. However, thrombosis may also lead to vascular occlusion and subsequent tissue damage.

In medicine, thrombosis is a hallmark of life-threatening cardiovascular diseases, such as myocardial infarction, stroke, or venous thromboembolism representing major causes of death in the Western civilization. Every year 17 million individuals die from cardiovascular disease (1, 2), comprising almost half of the global death toll in Europe. For example, in Austria, 34,000 patients suffered a cardiovascular death in 2012, accounting for 42.7% of all deaths, which is 1.7-fold the death toll of malignant diseases (3).

Cardiovascular science beyond clinical observation and anatomical dissection emerged in the late 19th and early 20th centuries. A key observation was the epidemiological connection of hypertension and elevated plasma lipids with atherosclerotic vascular disease (4). Based on a more profound understanding of these risk factors, including exercise, body weight, and glucose metabolism, preventive measures were tested, leading to both primary and secondary preventions that have become powerful modulators of disease decreasing events by almost half. Since then, the establishment of coronary care units, cardiac catheterization, angioplasty, and surgery and the advent of modern medications many of which interfere with thrombosis and platelet aggregation, have contributed to fundamental improvements in cardiovascular care. However, much of the advances in cardiovascular science have been in the thrombosis field, stimulated by the discovery that myocardial infarction was due to thrombi in the coronary arteries (5). Traditionally, thrombosis has been viewed as the biochemical result of regulated cascades of protein interactions characterized by the activation of factor X and the activation of thrombin, resulting in the formation of fibrin. Accordingly, treatments for myocardial infarction, stroke, and venous thromboembolism have targeted pathways or more recently, individual protein moieties (Figure 1). Table 1 summarizes landmark trials leading to the approval of individual compounds. Over 50 years, a plethora of treatments has been brought to market (Table 1), with high efficacy and safety profiles that have contributed to the observation that death rates per 100,000 population were decreased from 450 in the 1950s to 150 around 2010 (6). For example, while 20–30% of hospitalized patients with myocardial infarction died within 30 days due to their underlying disease, this number has been substantially decreased to <5% most recently (7).

**Figure 1:**
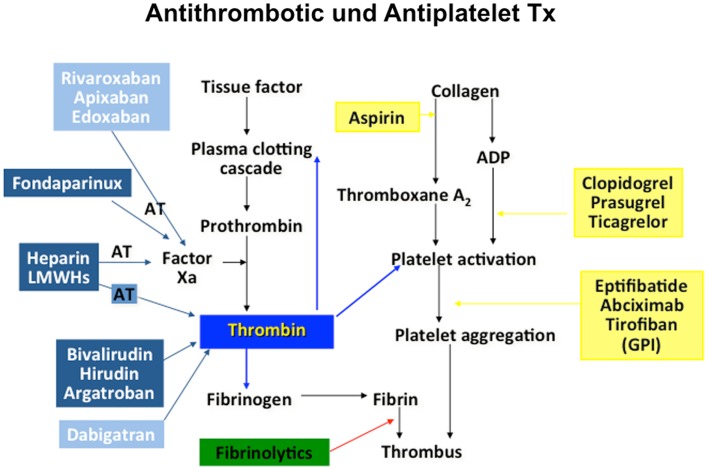
**Process of coagulation and the primary sites of action of current pharmacologic agents**.

**Table 1:** **Overview of treatments approved for vascular thrombosis (antithrombotics, comprising thrombolytics, anticoagulants, and antiplatelet drugs)**.

Concept	Mechanism of action	Compound (key reference)	Indication	Benefits/Harms
Antiplatelet drugs	COX inhibition	Acetylsalicylic acid/Aspirin (8)	Prevention of cardiovascular events	Increased risk of bleeding when combined with NSAIDs
	ADP receptor/P2Y_12_ inhibition	Clopidogrel (9)	Prevention of thrombotic events	Pro-drug limited by metabolization, irreversible
		Clopidogrel (10)	ACS	Pro-drug limited by metabolization, irreversible
		Prasugrel (11, 12)	Prevention of thrombotic events	Rapid onset of action, high efficacy, particularly in diabetic subjects, increased bleeding rates in those aged >75 years, and in those with previous stroke and a weight less than 60 kg, irreversible
		Ticagrelor (13)	Prevention of thrombotic events particularly in STE-ACS	Rapid onset of action, reversible, high efficacy, low bleeding rates, provides a survival benefit in ACS
	Phosphodiesterase inhibition	Cilostazol (14)	Reduction of symptoms of intermittent claudication	Phosphodiesterase 3 inhibitor, potentially dangerous in severe heart failure
	Glycoprotein IIb/IIIa inhibition	Abciximab (15, 16)	For use in individuals undergoing PCI with or without stent placement to decrease the incidence of ischemic complications due to the procedure	Effective prevention of ischemic events, particularly in diabetic subjects, and subjects with chronic kidney disease, increased bleeding rates only in high-risk patients
		Tirofiban (17)	Reduction of the rate of thrombotic cardiovascular events (combined endpoint of death, myocardial infarction, or refractory ischemia/repeat cardiac procedure) in patients with NSTE-ACS	Rapid onset and short duration of action (4–8 h)
		Eptifibatide (18)	Reduction of the risk of acute cardiac ischemic events (death and/or myocardial infarction) in patients with UA or NSTE-ACS both in patients who are to receive medical treatment and those undergoing PCI	Short half-life
Anticoagulants	Vitamin K antagonism	Vitamin K antagonists (19)	Prevention and treatment of venous thromboembolism, atrial fibrillation, mechanical and bioprosthetic heart valves, post-myocardial infarction, recurrent systemic embolism and other indications	Accepted standard, reproducible results, high exposure rates, cheap, no contraindication in patients with GFR <30 mL/min
	Factor Xa inhibition	Unfractionated heparin (20–23) new ESC Guidelines pending 2014	Deep vein thrombosis (DVT) in patients with renal failure	HIT possible
		Low-molecular weight heparins, e.g., enoxaparin (23, 24)	Prevention of DVT in hip or knee replacement surgery, or in abdominal surgery or acutely ill patients with severely restricted mobility at risk for thromboembolism	Accumulates in chronic renal failure
		Low-molecular weight heparins, e.g., enoxaparin (25, 26)	Prevention of ischemic complications of UA and NSTE-ACS. STE-ACS managed medically or with subsequent PCI	Accumulates in chronic renal failure
		Low-molecular weight heparins, e.g., enoxaparin (27)	Acute pulmonary embolism	Accumulates in chronic renal failure
		Fondaparinux (28)	ACS	Positive effect on survival, thrombus formation on wires/balloons during (primary) PCI if no additional heparin is used, contraindicated in severe renal failure with a GFR <20 mL/min
		Fondaparinux (29)	Prophylaxis of DVT in patients undergoing hip facture surgery, hip replacement surgery and knee replacement surgery.	50% VTE risk reduction compared with enoxaparin, no increase in clinically relevant bleeding Contraindicated in severe renal failure with a GFR <20 mL/min
		Rivaroxaban (30)	VTE prophylaxis	Easy administration, once daily, more effective compared with enoxaparin, same safety profile
		Rivaroxaban (31)	Non-valvular atrial fibrillation	Non-inferior to warfarin for the prevention of stroke or systemic embolism, same risk of major bleeding, however, less intracranial and fatal
		Rivaroxaban (32)	Reduction of the risk of recurrent atherothrombotic events in patients with acute coronary syndromes	Less cardiovascular death, myocardial infarction and stroke, but increased risk of major bleeding and intracranial hemorrhage, but not fatal bleeding
		Apixaban (33)	Non-valvular atrial fibrillation	Compared with warfarin less ischemic strokes
		Apixaban (34)	VTE prophylaxis	Superior to enoxaparin in preventing thrombosis
	Direct thrombin (II) inhibition	Lepirudin (35)	ACS	Low exposure rates, little information
		Bivalirudin (36)	ACS	Survival benefit, however, signal of increased early stent thrombosis
		Dabigatran (37), http://www.fda.gov/Drugs/DrugSafety/ucm396470.htm	Reduction of risk of stroke and systemic embolism in Non-valvular atrial fibrillation	Compared with warfarin less hemorrhagic strokes, mild increase of GI bleeds, antibody-based antidote in development
		Dabigatran (38)	Reduction of the risk of recurrence of DVT and pulmonary embolism	Compared with warfarin less hemorrhagic strokes, mild increase of GI bleeds, antibody-based antidote in development
		Dabigatran (39)	Treatment of DVT and pulmonary embolism	Compared with warfarin less hemorrhagic strokes, mild increase of GI bleeds, antibody-based antidote in development
		Argatroban (40)	Prophylaxis or treatment of thrombosis in patients with heparin-induced thrombocytopenia; including patients undergoing PCI	Low exposure rates, low case numbers
Thrombolytic drugs/fibrinolytics		Streptokinase (41)	Acute pulmonary embolism	Relatively low fibrin specificity, inexpensive
		Streptokinase (42)	Acute coronary syndrome	Relatively low fibrin specificity, inexpensive
		Alteplase (43)	Acute ischemic stroke	Recombinant tissue-type plasminogen activator with improved fibrin binding
		Reteplase (44)	Acute coronary syndrome	longer half-life, better penetration into thrombus
		Tenecteplase (45)	Reduction of mortality associated with acute myocardial infarction	higher fibrin specificity and greater resistance to inactivation by its endogenous inhibitor (PAI-1) compared to native t-PA

*Selected references and selected indications are displayed. COX = cyclo-oxygenase, NSAID = non-steroidal anti-inflammatory drugs, kg = kilograms, ACS = acute coronary syndrome, PCI = percutaneous coronary intervention, UA = unstable angina, STE-ACS = ST elevation acute coronary syndrome, NSTE-ACS = non-ST elevation acute coronary syndrome, GFR = glomerular filtration rate, HIT = heparin-induced thrombocytopenia, ACS = acute coronary syndromes, DVT = deep venous thrombosis, VTE = venous thromboembolism, GI = gastro-intestinal, PAI-1 = plasminogen activator inhibitor type 1, t-PA = tissue-type plasminogen activator*.

The public domain is thrilled with advances of cardiovascular medicine, and needs for deeper insights are hardly convincing in an environment of harmonized health care providing infarction networks, stent-for-life programs, and generally high standard guidelines in cardiovascular care.

However, do we understand thrombosis, do we understand recurrence, or do we prefer, for example, to mandate life-long factor X inhibition (38, 46–48) and statins (49) in patients at risk, without further investigation of underlying mechanisms?

More recently, a new view of thrombosis has emerged accounting for thrombosis as a vascular disease involving the dynamic interaction between platelets, circulating inflammatory cells, nucleic acids and proteins, and resident cells of the vascular wall. Milestones along the way of integrating the vessel wall in the thrombotic process were the discovery and action of nitric oxide (50, 51), the delineation of the LDL-cholesterol pathway (52, 53), the role of soluble and membrane bound tissue factor in atherosclerosis (54), and most recently the concept of “immunothrombosis” (55), linking inflammation and thrombosis as regulators of vascular integrity. Consequently, it was understood that the hemostatic system is a modulator of atherosclerosis (56). Thrombosis comprises both acute clotting and the more time-consuming process of thrombus resolution, which represents a vascular remodeling process that is driven by inflammation, angiogenesis, and cells of the innate immune system. A stimulus leading to thrombus formation induces an innate immune response that is supported by neutrophils, lymphocytes, macrophages, by specific thrombosis-related molecules, and neutrophil extracellular traps (NETS), underpinning the importance of the following questions:

Is thrombosis the underlying process behind both arterial and venous disease that oftentimes combines in individual patients? (57) Do we understand endothelial dysfunction, and is it the nidus for thrombosis, and eventually vascular occlusion? And why do acute pulmonary emboli transform into chronic vascular obstructions in chronic thromboembolic pulmonary hypertension? Do we understand acute vascular syndromes simply by plaque rupture that is driven by macrophages that are loaded with lipids, and proteolytically cleave a thin-cap fibroatheroma thus triggering platelet activation? Does that concept explain acute coronary syndromes in young subjects without significant vascular stenoses? Is thrombosis *per se* the trigger for all vascular occlusion, be it chronic or acute? Is vascular occlusion the sequlae of deficient efferocytosis, and are those mechanisms driven by a deficiency of natural antibodies? What are the recognition sites for natural antibodies in the circulation? Which cells or cell fragments in the circulation are carrying oxidation specific epitopes, e.g., oxidized low-density lipoprotein (OxLDL) or malon-dialdehyde, and how do they function as danger associated molecular patterns? What is the role of nucleic acids, RNAse, and DNAse in thrombosis? Is NETting a preventable amplifier of acute thrombosis and the adverse vascular remodeling following thrombosis?

Are there potential therapeutic targets, upstream of mechanisms of thrombosis that might be applied without any potential bleeding risk? And might addressing those new targets prevent recurrence? Will we be able to identify a cellular program to minimize the potential of thrombosis and vascular occlusion? Will we be restoring normal Ca^2+^ cycling and reduce reperfusion injury? Will we be utilizing therapeutic antagomirs to control cellular responses to vascular injury? Will we be engineering vessels and vascular compartments of organ systems?

While bench-top research will address these questions, clinical research will complement advances both in drug-based trials and in interventional approaches. Since the PEITHO trial, the term of acute pulmonary revascularization has been coined in analogy to coronary revascularization, leading the way to interventional treatments (58) as adjunct or stand-alone treatments for the pulmonary circulation. Similar developments have recently been initiated in chronic pulmonary vascular disease and will change treatment paradigms (59–62).

Taken together, thrombosis is an important mediator of vascular disease, but cannot be seen in isolation. Data suggest that it most often occurs as the consequence of a loss of vascular barrier function, for example, in the context of inflammation and rarely as a primary event. To comprehend mechanisms of vascular barrier function is another challenge of thrombosis research of the future. To understand thrombosis, we need to look beyond thrombosis.

## Conflict of Interest Statement

Irene M. Lang has relationships with drug companies including AOPOrphan Pharmaceuticals, Abbott, Actelion, Astra-Zeneca, Bayer-Schering, Cordis, Glaxo Smith Kline, Medtronic, Novartis, Pfizer, Servier, and United Therapeutics. In addition to being investigator in trials involving these companies, relationships include consultancy service, research grants, and membership of scientific advisory boards.
